# Eruption of a deep-sea mud volcano triggers rapid sediment movement

**DOI:** 10.1038/ncomms6385

**Published:** 2014-11-11

**Authors:** Tomas Feseker, Antje Boetius, Frank Wenzhöfer, Jerome Blandin, Karine Olu, Dana R. Yoerger, Richard Camilli, Christopher R. German, Dirk de Beer

**Affiliations:** 1 MARUM—Center for Marine Environmental Sciences and Faculty of Geosciences, University of Bremen, Bremen 28359, Germany; 2 GEOMAR, Helmholtz Centre for Ocean Research Kiel, Kiel 24148, Germany; 3 HGF-MPG Group for Deep Sea Ecology and Technology, Alfred Wegener Institute, Bremerhaven 27515, Germany; 4 Max Planck Institute for Marine Microbiology, Bremen 28359, Germany; 5 Ifremer, Institut Carnot EDROME, RDT/SI2M, Plouzané F-29280, France; 6 Ifremer, Institut Carnot EDROME, REM/EEP, Laboratoire Environnement Profond, Plouzané F-29280, France; 7 Woods Hole Oceanographic Institution (WHOI), Woods Hole, Massachusetts 02543, USA

## Abstract

Submarine mud volcanoes are important sources of methane to the water column. However, the temporal variability of their mud and methane emissions is unknown. Methane emissions were previously proposed to result from a dynamic equilibrium between upward migration and consumption at the seabed by methane-consuming microbes. Here we show non-steady-state situations of vigorous mud movement that are revealed through variations in fluid flow, seabed temperature and seafloor bathymetry. Time series data for pressure, temperature, pH and seafloor photography were collected over 431 days using a benthic observatory at the active Håkon Mosby Mud Volcano. We documented 25 pulses of hot subsurface fluids, accompanied by eruptions that changed the landscape of the mud volcano. Four major events triggered rapid sediment uplift of more than a metre in height, substantial lateral flow of muds at average velocities of 0.4 m per day, and significant emissions of methane and CO_2_ from the seafloor.

## Abstract (web summary)

Submarine mud volcanoes are difficult to observe from the sea surface and previous recordings at depth have been short term. Here, the authors provide the first long-term monitoring from Håkon Mosby and suggest that mud volcanoes may be more important to the global methane budget than previously thought.

## Graphical abstract

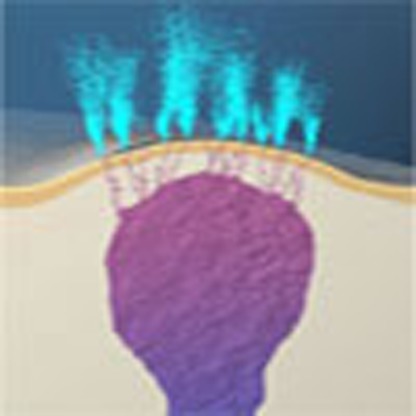

Submarine mud volcanoes are geologic structures deeply rooted into the subsurface seafloor, formed by mud expulsions and associated transport of warm, deep-sourced fluids and gas, predominantly methane^1^,^2^. At high pressures and low temperatures, methane oversaturation can result in gas hydrate formation in sediment pore spaces, which may seal the seafloor against upwardly migrating fluids^3^. Gas hydrates, hence, may act as a buffer, providing a persistent source of dissolved methane to near-surface sediments and fuelling rich and diverse ecosystems^4^,^5^. Anaerobic and aerobic microbial oxidation consume the majority of the methane advected to near-surface sediments, where sulphate and oxygen are available as electron acceptors^6^,^7^. However, this microbial filter is less efficient when the ascent rates of subsurface fluids are high, as the flux of electron acceptors (for example, sulphate) into the sediments decreases^1^,^8^. At very high fluid-flow velocities, bubble-forming gaseous methane can bypass the microbial filter within the sediments completely and escape into the overlying water column^9^,^10^. Dissolved methane discharge from the seafloor can also occur when rapid seepage hinders the penetration of seawater-derived electron acceptors into the sediment^11^ or when methane-oxidizing microorganisms are absent from freshly erupted mud^1^. Consequently, when seepage is vigorous and/or when mud is remobilized, the proportion of the total methane flux being oxidized by the microbial filter may be <40% (refs 7, 12), depending on upflow rates and frequencies of disturbance. The total methane emission from offshore mud volcanoes into the atmosphere has been estimated at 27 Tg per year^13^, but this value has large associated uncertainties, because the total number of mud volcanoes worldwide and their temporal variability with respect to methane emissions are both unknown. Various historical records suggest that eruptions of onshore mud volcanoes occur with intervals of years to decades^14^,^15^. Marine mud volcanism can also be episodic^16^, but the characteristics and frequency of eruptions of deep-sea mud volcanoes are not yet documented. To investigate the temporal variability of deep-sea mud-volcanic activity, we deployed a seafloor observatory for a period of 431 days in 2009–2010 at the active Håkon Mosby mud volcano (HMMV), located at 1,250 m water depth on the Barents Sea slope^17^.

The HMMV is a circular feature of around 1 km in diameter with a shallow relief of <10 m above the surrounding seafloor. Extremely high geothermal gradients of >25 °C m^−1^ in the upper metre of the seafloor of the northern part of the mud volcano’s centre indicate high upward fluid flow rates^18^ of >4 m per year above the deeply rooted mud volcano chimney^17^. There, a central, flat area of soft, rippled muds is surrounded by more consolidated muds covered by bacterial mats, and a hummocky ring of hydrate-bearing mounds covered by siboglinid tubeworms^19^. The concentric structure of these habitats has been attributed to a gradient in seepage rates^8^, from the central upflow pipe to the outer rim, of 5–<0.1 m per year. Across the mud volcano and particularly in the hummocky periphery, gas hydrates are abundant, and gas ebullition from the seabed has been observed frequently, resulting in large gas flares that can be detected readily in the overlying water column^17^.

In a large interdisciplinary effort we designed, built and deployed a device for long-term observations on mud-volcano eruptions (LOOME). The objective of the LOOME observatory was to derive a timeline of temperature gradients and mud transport, combined with seafloor observations that would allow us to test the hypotheses that temperature dynamics, morphological and bathymetrical changes could be related to eruptions of gas and mud^20^.

Comparisons of maps made during this project and previous research cruises showed drastic topological changes developing over years. Our LOOME observatory recorded even more substantial sediment and fluid dynamics, occurring over days to weeks. Our data provided a basis for a mechanistic understanding of the geological and geochemical activity of this large mud volcano. We conclude that the amount of escaping methane is much larger than previously thought.

## Results

### Deployment and site

**Figure 1:**
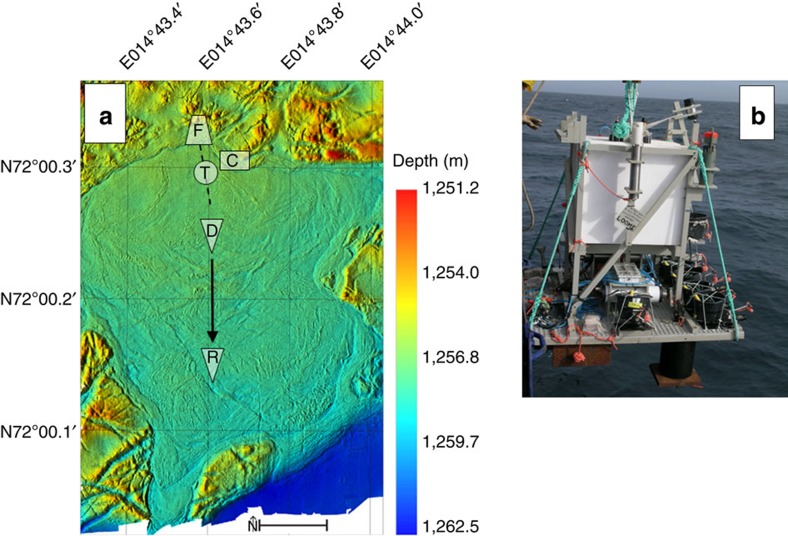
Overview of the deployment area and instruments. (**a**) Shaded relief map of the central area of the Håkon Mosby mud volcano. The LOOME observatory frame (F) was positioned north of the centre, where the hummocky seafloor provided a solid base for our long-term instrument deployment. The seabed thermistor chain (dashed line) with 24 sensor nodes was laid out across the northern part of the active centre. The short temperature lance and the chemistry logger (T) were placed at the position of the highest geothermal gradient. The camera (C) was placed on the edge of the central area in a field of abundant microbial mats. The long temperature lance was deployed south of the centre in July 2009 (D) but had moved 165 m further to the south by the time it was recovered in September 2010 (R). Scale bar, 100 m. (**b**) The LOOME observatory frame before deployment. The black buckets contained the cables by which the sensors were connected to data loggers mounted on the frame.

The LOOME observatory frame with data loggers was deployed in July 2009 within the peripheral hummocky area. Using a remotely operated vehicle, the observatory was positioned precisely on top of a stable slab, to avoid data loss in the event of any destructive eruptions that might damage the attached instrumentation deployed across the interior of the HMMV ‘caldera’. The location of the frame (Fig. 1; Supplementary Table 1) was ~50 m north of the active centre, of the HMMV that is characterized by high temperature gradients (Supplementary Fig. 1). The remotely operated vehicle (ROV) *QUEST* deployed three cables from the LOOME observatory frame that extended out into the active centre, each with distinct scientific instrumentation connected back to the central data loggers on the LOOME frame: (1) an 80-cm-long sediment temperature lance; (2) a pH sensor and (3) a temperature sensor chain with 24 sensors distributed over 100 m (Fig. 1). A CTD system (recording conductivity, temperature and density; Seabird, SBE 911plus) equipped with an additional pressure sensor, as well as a recording current meter with a turbidity sensor were mounted on the LOOME frame. Two further independent instrument deployments were implemented as part of our LOOME observatory efforts: (4) the AIM autonomous underwater video camera^21^ was installed at the transition facing towards the active centre in an area of the seafloor covered by abundant microbial mats; (5) a 12-m-long sediment temperature lance was deployed at the southern transition between the active centre and the surrounding muds. Surveys by an autonomous underwater vehicle (AUV), provided continuous spatial observations complementing shipboard observations in 2010.

### Vertical movements

**Figure 2:**
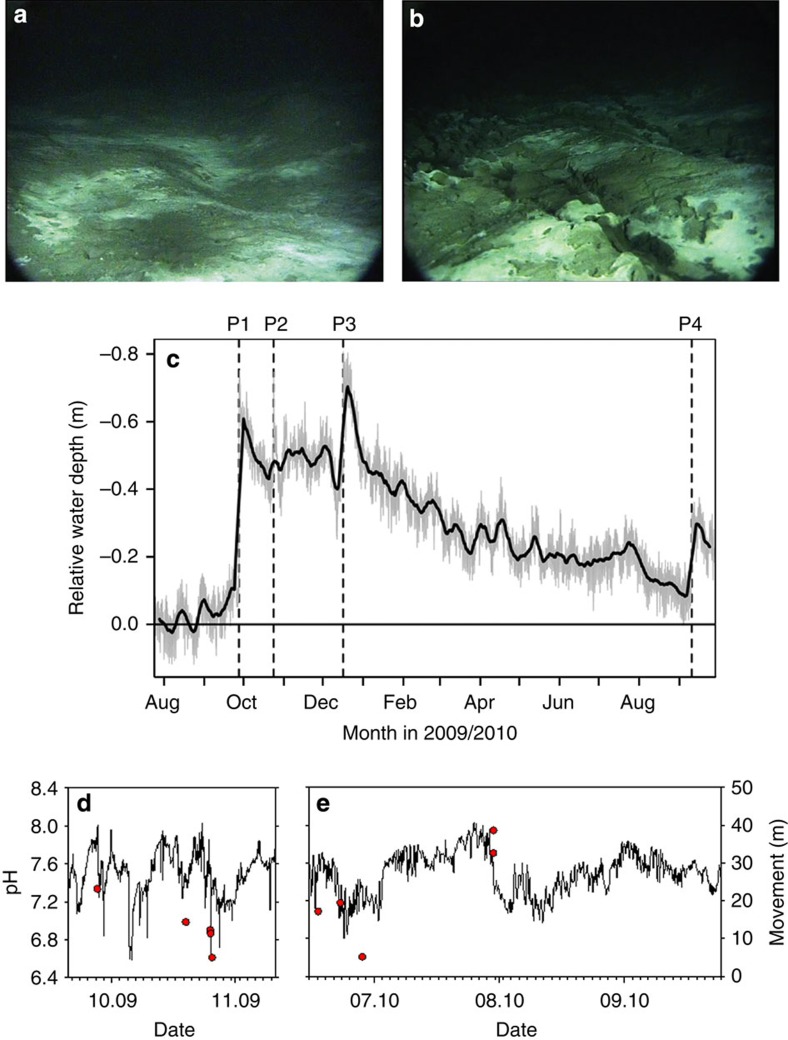
Observations of seafloor disturbances. Still images taken from video footage collected by the AIM seabed camera system recorded on 27 September 2009 at (**a**) 08:58 and (**b**) 20:58 UTC (copyright Ifremer-AIM). Field of view in parts (a) and (b) is approximately 1m. Field of view in parts (**a**) and (**b**) is approximately 1 m. (**c**) Time series of water depth at the location of the LOOME observatory frame (Fig. 1). Variations in bottom-water pressure revealed four rapid uplifting events (P1–P4), which were each followed by slower subsidence. The solid black line presents the running mean for a time-window of 1 week. (**d**,**e**) Drift-calibrated *in situ* pH dynamics (see Methods section), the red symbols indicate timing and distance of sediment movements (Supplementary Table 4).

Bottom-water pressure data recorded at the observatory frame, converted into a time series of water depth at the seafloor, indicated four major events during 2009 and 2010, as defined here by a sudden uplift of the seabed at rates between 0.2 and 0.7 m h^−1^ (Fig. 2c; Supplementary Table 2). The most pronounced uplift event, P1 (*ca.* 0.6 m over 3.5 h), started at 12:40 UTC on 27 September 2009. A comparison of images recorded by the underwater video camera pointing towards the central mud area at 08:58 UTC and at 20:58 UTC (27 September 2009) clearly shows that the seafloor ruptured, indicative of deformation associated with the uplift event P1 (Fig. 2a,b). During the following 9 months, three further but smaller uplift events were recorded. The images did not show resuspension of sediments in the form of particle clouds. Likewise, the turbidity sensor did not record immediate disturbances at the time of uplift (Supplementary Table 3). Cumulatively, the four major events resulted in a total uplift of 1.3 m, but the seafloor also deflated gradually in between each of those events such that, by August 2010, the seafloor had gained only 0.2 m in increased height compared with the September 2009 start of our time series.

### Horizontal movements

These events, all recorded at the relatively stable hummocky area to the north of the active centre, were accompanied by drastic evidence of seafloor dynamics in the centre of the HMMV. A short temperature lance had been inserted vertically into the sediment at a location 60 m south of the frame, where we expected the highest level of volcanic activity. When we inspected the instrument on 27 September 2010 prior to its recovery, the lance had been pulled out of the sediment and was lying horizontally on the seafloor (Supplementary Note 1). On 26 October 2009, two days after the sudden uplifting event P2, the temperature signals for all of the sensors along the length of that lance had decreased to bottom-water temperature levels over a period of <3 h (Supplementary Fig. 1). We interpret these results to indicate the time and date for the onset of lateral sediment displacement at the centre of the HMMV which, because the lance was tethered to the fixed LOOME observatory frame, had been pulled out of the laterally displaced mud until it came to rest on the HMMV surface. Further support for this inference comes from our separate visual observation that the similarly cable-tethered buckets housing the pH logger and temperature sensors attached to the LOOME observatory frame had left similar parallel trails in the surface of the sediment, as if they had been pulled across the seafloor. The cables connecting the various sensors to the logger on the LOOME observatory frame were all fully stretched (Supplementary Fig. 2). Perhaps most surprising of all, however, was the observation that our 12-m-long temperature lance had been relocated to ~165 m south of its deployment position by the time it was recovered (Fig. 1; Supplementary Fig. 3). This lance was deployed in 2009 at the southern transition of the active centre of the HMMV, where it had sunk deep into the soft muds to about 17 m below the seafloor. Thus it was transported laterally with a mean velocity of ~0.4 m per day over the course of the 431-day deployment. The displaced lance remained upright (vertical) relative to the seafloor and operated thoroughly throughout its transit, successfully recording substantial cooling of initially hot surface sediments above a warm (maximum temperature 23 °C) layer of mud (Supplementary Fig. 4).

### Episodic horizontal movements

**Figure 3:**
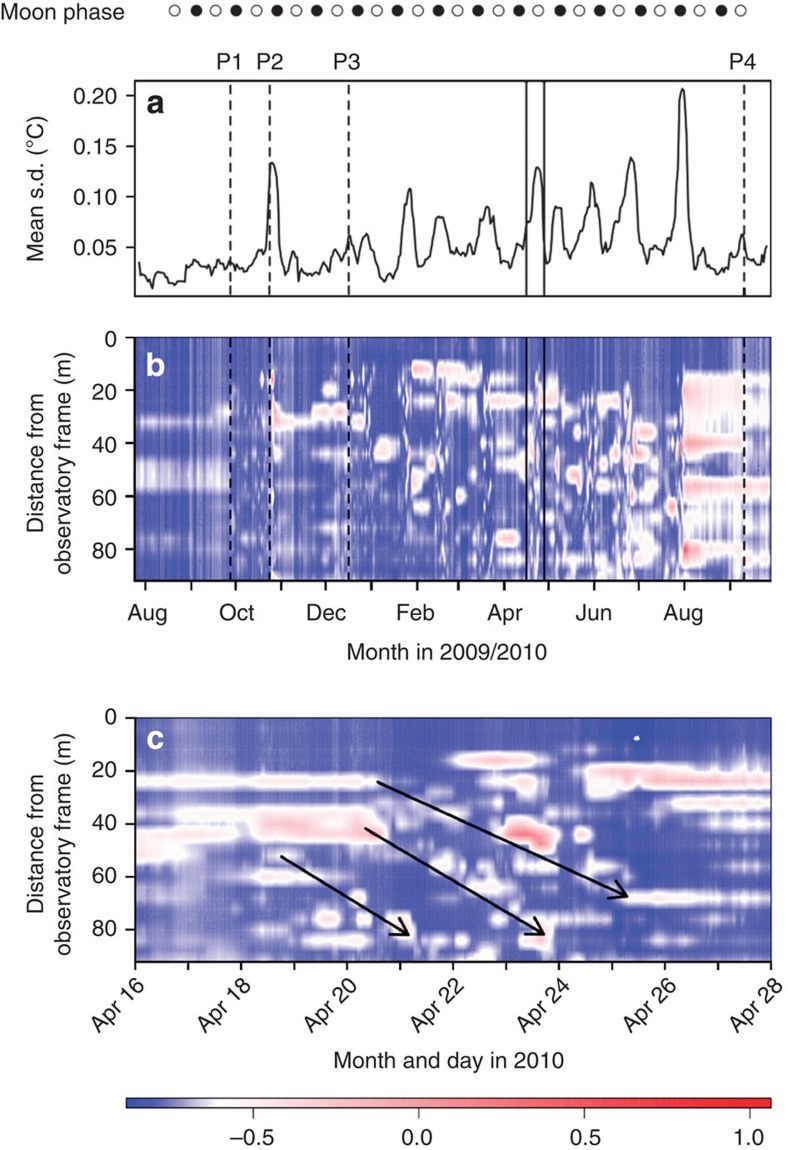
Time series of seabed temperature across the central area of the HMMV. (**a**) Mean running s.d. of seabed temperature (24 h mean) during the entire observation. P1 to P4 refer to the four major uplifting events. (**b**) Contour plot of the seabed temperature (24 h mean) along the thermistor chain during the deployment time. Blue colours represent the normal bottom-water temperature range (−0.9 to −0.7 °C), while red colours show seabed temperatures >−0.7 °C, attributable to mud volcanic activity. The latter are only found 12 m or more from the frame. (**c**) High-resolution contour plot of seabed temperature during a time interval of 12 days in April 2010. The arrows indicate traces of warm sediment, which are interpreted as sediment movement.

The seabed thermistor chain that covered a 100-m-long N–S transect from the LOOME observatory frame across the hot central area recorded multiple transient local temperature anomalies (Fig. 3). The three sensors located outside the active central area near the observatory frame (T22–T24) recorded background seafloor temperatures between −0.886 and −0.657 °C, close to those of local bottom water, throughout the entire time series. In contrast, at the positions of the other sensors (T1–T21), temperatures ranged between −0.057 and 10.931 °C. Distributions of temperature anomalies observed along this temperature chain are consistent with a progressive migration away from the LOOME observatory frame, that is, southwards away from the active centre over the course of our time series records. Plots of these temporal dynamics in surface seabed temperature along the thermistor chain (distance of 100 m) reveal a diagonal trend for these anomalies that we interpret as lateral transport of warm mud (Fig. 3c; Supplementary Fig. 5). Some of the pronounced anomalies were sustained along the whole length of the string, and indicate a displacement of 160–200 m over the observation period, providing close independent agreement with the ~165-m displacement observed for the 12-m T-lance (see previous section). Close inspection of Fig. 3 reveals that this ~165 m of movement was not continuous and gradual but, rather, occurred as distinct episodes, separated by longer, static periods (Fig. 3b,c). In total, we have identified evidence for 25 flow events, grouped into 13 distinct episodes of sediment movement that each lasted for periods from 4 h up to ~8 days (see Supplementary Table 4). Some of these episodes coincided with phases of elevated turbidity signals (Supplementary Table 3; Supplementary Note 2). The sum of the average displacements achieved over these 13 episodes, cumulatively, was 280–350 m (sum of the largest distances of each period from Supplementary Table 4; Supplementary Fig. 5), that is, even further than the observed displacement of the 12-m T-lance. Thus, the lateral sediment velocity during the entire observation averaged about 0.5–1 m per day across the active centre.

During several displacement events the pH at the surface decreased, indicating that mud movement was accompanied by release of warm CO_2_-rich subsurface fluids (Fig. 2d,e). Indeed, both methane and CO_2_ flares were observed in the most active area by the AUV *Sentry* equipped with the *in situ* mass spectrometer TETHYS (Supplementary Fig. 6). Unsurprisingly, the spatial distributions of gas flares resulting from ebullition of gas bubbles from the seafloor had changed between 2009 and 2010. Between 2009 and 2010 the position of these flares showed an overall shift in southeasterly directions (Supplementary Fig. 7). Seafloor photography of the active, warm centre showed conspicuous cracks and troughs as well as distinct mud flows (Supplementary Fig. 3) while similar surveys of the southward region revealed that the seafloor was littered with small holes, consistent with the hollow depressions left behind after gas bubbles had been extruded through and released from these soft, warm sediments (Supplementary Fig. 8).

**Figure 4:**
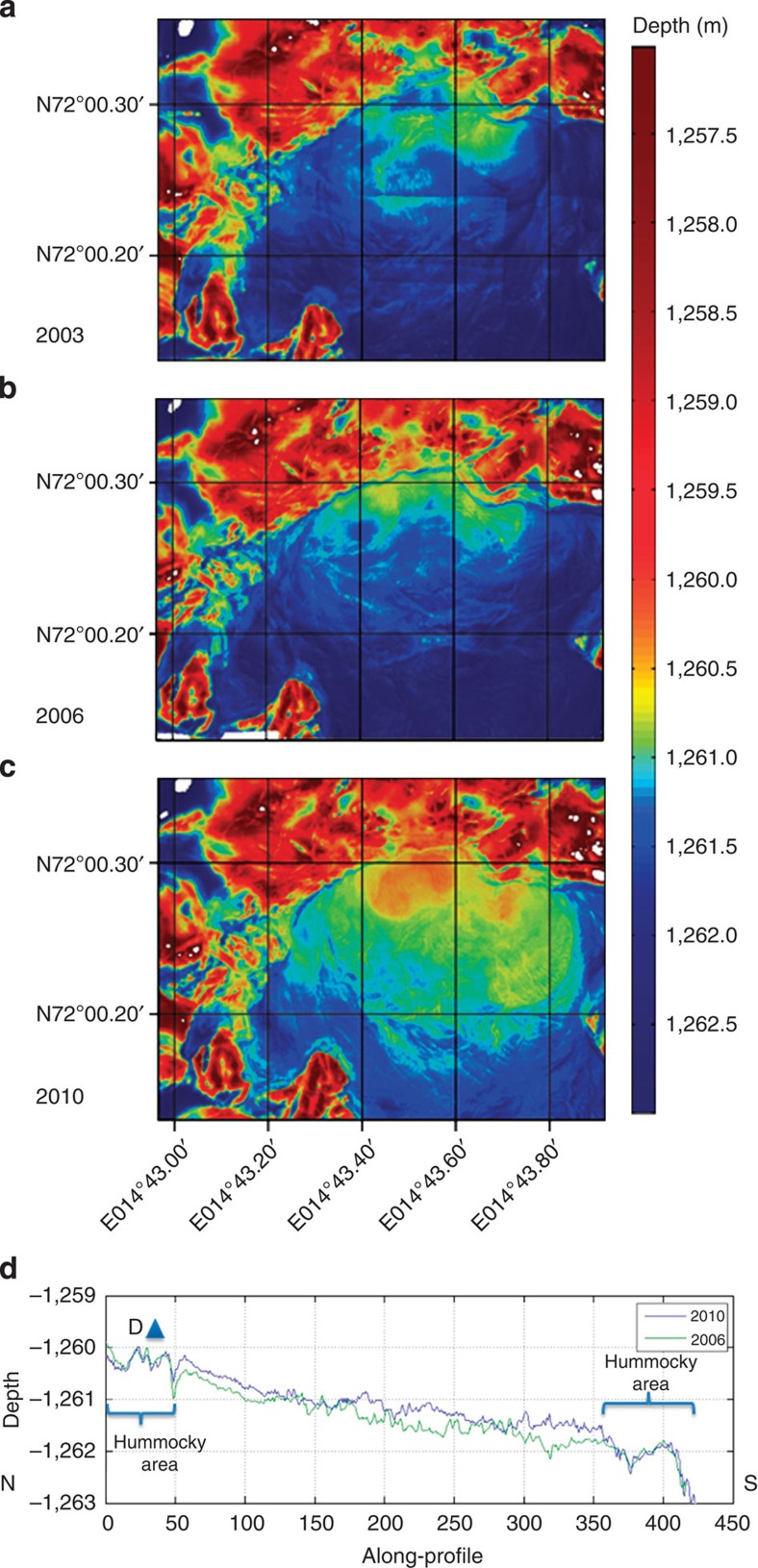
High-resolution bathymetric maps. Maps from (**a**) 2003, (**b**) 2006 and (**c**) 2010 show subtle changes in seabed topography. (**d**) A comparison of the 2006 and 2010 N–S cross-sections over the HMMV Central Zone close by the site of the LOOME observatory frame (from 72° 0.345'N, 014° 43.530'E to 72° 0.023'N, 014° 43.530'E) reveals a net uplifting of the surface of the central area by 0.0–0.6 m (average 0.3 m) over the 4-year period. Multibeam data were collected by ROV Victor (2003, 2006; ref. 20) and AUV *Sentry* (2010; this study). Microbathymetry profiles were matched by assuming that the depth of the hummocky zone rimming the central HMMV structure remained constant.

High-resolution bathymetric maps made in 2003 (Ifremer), 2006 (Ifremer) and 2010 (WHOI) indicate that the mud volcano centre, and especially its warm, elevated northern part, has been dynamic over the past decade^20^ (Fig. 4a–c). Comparison of extracted morphologies from north to south across the mud volcano reveals an irregular increase in height of 0–0.6 m with, in the most active area (50–150 m along profile), an increase of ~0.25 m (Fig. 4d).

## Discussion

Our 1-year deployment of independent sensors recorded episodes of massive lateral mud transport related to the upward flow of hot fluids. The very rapid uplift and slower deflation of the seafloor below the observatory can only be explained by rapid expansion of trapped gasses followed by their gradual release, causing the observed disturbances of seafloor morphology. This is further corroborated by the constant temperature in the area close to the observatory frame, confirming that the lifting and cracking of the surface seafloor in this area was caused by gas expansion. Further away, in the active central zone of the HMMV, which is characterized by mobile muds, seabed temperatures peaked markedly during sediment movement, indicating that considerable local seabed warming occurred due to mud-volcanic activity transporting warm fluids. The 24-h mean seabed temperature record over the entire observation period revealed elevated temperatures, in particular, during episodes of active sediment movement (Fig. 3a). These mud displacements occurred on a roughly monthly basis, but were not linked to the lunar phase as has been suggested previously for terrestrial mud volcanoes^14^.

Our findings show for the first time the sequence of landscape-scale changes of a deep-sea mud volcano and, together with previous observations, support the hypothesis of recurring episodic degassing of the HMMV^17^,^18^,^20^. Previous recordings in 2005–2006 of the temperature near the active area showed sudden cooling events in the upper 15 m, and short temperature sensors deployed by 2005 in the active central area had completely disappeared in 2006 (ref. 22). The microbathymetry maps of 2003, 2006 and 2010 show changes in morphology, especially in the active northern centre (Fig. 4a–c), indicative of mud movements. Interestingly, comparison of those maps reveals no evidence of sediment overflow to the surrounding area, even in the flat south-eastern edge of the HMMV (Fig. 1; see also maps in Foucher *et al*.^20^). In summary, such mud movements must be episodically recurring events, yet there are no signs of massive accumulations of mud in the HMMV, and there is no evidence of mud flow to the outside of the volcano. Instead, volume is apparently conserved which, consequently, requires that the massive upward and lateral mud movements that we have recorded must be accompanied by an equal and opposite recycling of mud volume within the HMMV mud chamber^17^, for example, through the sinking of degassed and cooled sediment layers (Supplementary Fig. 4)—which, therefore, would be relatively dense—at the outer periphery of the HMMV.

Seismic signatures recorded in 2005 and 2006 suggest that transport and expansion of methane can cause a significantly lower density in the deep subsurface mud chamber fuelling the central chimney of the mud volcano when compared with the overlying and surrounding sediments^17^. A temporary increase in gas supply from the root of the HMMV could lead to unstable density gradients, and a rapid upward transport of warm, gassy muds. Such events could dissociate the gas hydrates that are usually present at the surface of the HMMV as it is cooled by the overlying cold bottom water. The slope of the HMMV surface has an overall gradient of 0.3 m per 100 m. While hydrates should stabilize the surface muds during quiescent periods^23^,^24^, any hydrate destabilization and expansion of gas by warming would be expected to lead to a substantial bulging of the seafloor, which could then, in turn, result in the lateral sliding of mud down-slope from north to south. Such a mechanism would explain both the southward movements of warm muds as recorded by the thermistor chain and the displacement of the thermal lance. The lance was positioned vertically into the sediment over a depth horizon of 8–20 m below the seafloor during the whole deployment period which, therefore, requires that a consolidated slab of mud of at least 20 m in thickness was displaced southward, intact, over a distance of at least 165 m. This, in turn, must have been resupplied by fresh, gassy mud expelled at the centre of the active zone. Comparing maps generated in 2006 with those acquired during the recovery cruise in 2010, we estimate the width of the mobile area to be ~200 m (Fig. 4d), equivalent to a mobile volume of the order of 660,000 m^3^ of gassy mud (calculated as 20 × 165 × 200 m). Extrapolating the overall uplift of the seafloor bathymetry (Fig. 3) by an average of 0.3 m between 2006 and 2010 to the entire centre area of the mud volcano (200,000 m^2^) results in a volume increase in 4 years of only 60,000 m^3^, that is, <10% of the estimated volume that moved in 2009–2010. We do not exclude the possibility that processes active in the volcano could also expel subsurface porewaters, which would reduce the estimated amount of gas required to elevate the sediments. Indeed, the pH anomalies recorded in the HMMV sediments argue for an enhanced expulsion of rather acidic porewater^7^,^8^ during sediment movement. While porewater flow in the central area can reach upward flow velocities of several metres per year^2^,^8^,^18^, the uplift we have recorded here was much faster, up to 60 cm in a few hours—that is, three orders of magnitude faster than porewater movements could achieve. Consequently, we conclude that most of the uplift we report here must have been gas driven.

Over the entire period four very rapid uplift events were recorded, totalling 1.3 m, accompanied by release of gas from the sediment and each followed by seafloor subsidence. It should be noted that the active central HMMV area may have experienced even more uplift than recorded at the location of the LOOME observatory frame situated on the hummocky terrain at the Northern edge of the HMMV. For example, Fig. 4 shows that areas 175–350 m south of the frame became more elevated than the hummocky area, where the frame was positioned and where the vertical dynamics were registered. With the assumption that these events were mostly gas driven, we can estimate a gas expansion of 1.3 m^3^ m^−2^ of seafloor. Most of this gas was likely supplied through upward fluid advection from the deep subsurface, but in part it may also have originated from destabilization of gas hydrates located within the upper sediment layers. Applying this unit volume of gas expansion and extrapolating it to cover the whole of the displaced/elevated mud area (165 × 200 m), would imply a release of 43,000 m^3^ of methane at 120 atm (the *in situ* pressure at the seafloor at the HMMV), which is equivalent to 2 × 10^8^ mol of methane gas. In earlier work it was estimated that 8–35 × 10^6^ mol of methane escape from HMMV per year in the form of gas bubbles released from single streams^25^, an estimate that does not include recurrent eruptions of the kind documented here. For example, if eruptions were to occur at a frequency of one eruptive event per year with the same magnitude that we describe here, the total annual methane release from the HMMV mud volcano would be an order of magnitude higher than all previous estimates. In comparison, the sum of the biological removal rates for methane released at HMMV, by aerobic and anaerobic oxidation, is only 5–15 × 10^6^ mol per year^7^. Thus, from our new calculations, we would predict that <3% of the total methane released from the HMMV might be oxidized at or beneath the seafloor. Thus the vast majority of the methane released escapes into the overlying water column and, potentially, the atmosphere^20^,^24^,^25^.

Of course, we cannot be completely certain of the total area of seafloor uplifted, whether the average elevation of 1.3 m reported here is typical of such an event, and what the frequency of recurrence might be for the events observed here. Consequently, our calculations on the amounts of released gas must be considered as an indicative/illustrative first approximation. However, our core conclusions—that much more methane gas is released from the HMMV than had previously been appreciated and that only a very small fraction of the total methane flux might be microbially mediated and mitigated in the upper sediments—agree closely with the identical isotope compositions observed for methane both within HMMV hydrates and in dissolved methane sampled in the overlying watercolumn^24^. Moreover, the sporadic extrusion of subsurface muds onto the mud volcano centre is likely to create major disturbances to the benthic communities present, as evidenced from the seabed photography surveys completed during our mission (Supplementary Fig. 3). Such perturbations to these ecosystems will inhibit the development of efficiently methane-consuming microbial communities in the area of highest methane fluxes. Because anaerobic methane-oxidizing consortia only grow very slowly, re-establishing such a microbial filter on freshly expelled mud may require months to years—that is, over timescales that may be comparable to or even longer than the intervals anticipated between successive eruption events^26^. Therefore, we hypothesize that recurrent eruption events at deep-water mud volcanoes such as HMMV may represent significant previously overlooked sources for dissolved methane release to the hydrosphere; these require further evaluation through continuous long-term observations.

## Methods

### Observatory design

The LOOME observatory comprised sensors for the subsurface, the seabed and the water column. The frame was a platform measuring 2 × 2 m, which carried two CTDs, a horizontal-looking sonar and a recording current meter with a turbidity sensor (Aanderaa, Norway). The current meter data showed that the LOOME frame was positioned upstream of the active centre (Supplementary Table 3). Data loggers for the short temperature lance, the seabed thermistor chain and six chemical sensors including pH were mounted on the frame. After the deployment by winch on 24 July 2009 during the R/V *Polarstern* mission ARKXXIV-2, the observatory was positioned precisely using the ROV *QUEST* (MARUM), and the cabled sensors were installed at selected locations in the central area of the HMMV (Fig. 1). The AIM underwater camera (Ifremer) and the long temperature lance (Ifremer) were autonomous instruments, and deployed separately, the camera by ROV and the temperature lance by the ship winch. The observatory frame and the camera were recovered on 28 September 2010 during the R/V *Maria S. Merian* expedition MSM16/1 by connecting the ship’s wire to the instruments using the ROV *Genesis* (Ghent University). The long temperature lance was recovered in the same way on 30 September 2010. Upon recovery of the LOOME observatory frame it was discovered that one of the two CTDs, the horizontal-looking sonar and five of the six chemical sensors had suffered from technical failures and had not recorded any data. All positions of the instruments and their metadata are deposited at the international Earth system data base PANGAEA^27^. Supplementary Table 1 shows the deployment and recovery positions of the LOOME observatory instruments discussed here.

### Pressure and seafloor elevation

Pressure time series at the position of the LOOME frame were obtained independently from pressure sensors integrated into the CTD and into the data logger connected to the short temperature lance, respectively. The sampling interval was 20 min for both instruments. The two sensors showed a mean offset of 16.2 dbar, which was attributed to a calibration offset of the CTD. The deviations of the offset-corrected data ranged between −0.12 and 0.13 dbar. The analyses shown here were based on the data obtained by the Keller PA 8 200 pressure sensor integrated into the data logger, which was calibrated to a precision of ±0.05 dbar prior to deployment.

After subtracting the standard atmospheric pressure of 1,013.25 mbar, the pressure time series was converted to depth^17^ for the latitude of 72°N and detided by subtracting tide heights modelled for the position of the HMMV using the TPXO7.1 global inverse tide model^18^. The resulting depth time series ranges between 1,256.7 and 1,257.6 m with a mean depth of 1,257.2 m, which is in reasonable agreement with the water depth at the position of the LOOME frame of 1,256.3 m, according to the micro-bathymetric map from 2006 (ref. 19).

### Underwater video camera

The deep-sea video camera AIM (Ifremer)^21^ was programmed to record two video clips of 2 min each per day. The battery was depleted in December 2009. For Fig. 2, two stills were extracted from the videos 08:58 and 20:58, 27 September 2009.

### Long sediment temperature lance

On 26 July 2009, we deployed a 12-m-long gravity corer equipped with autonomous temperature loggers mounted on outriggers to obtain *in situ* sediment temperature measurements from deeper layers of the mud volcano. The total weight of the instrument including the weight on the corer head is ~1,000 kg. A 20-m-long rope with a buoy was attached to the corer head to facilitate the recovery of the instrument. Connected to the ship’s wire via an acoustic release, the instrument was lowered until a decrease of the load indicated that it had penetrated the seafloor, then it was released. The positioning was controlled via an Ultra Short Baseline system (USBL) and verified by ROV. The entire lance sank into the muds to a depth of 20 m, so that only the rope with floatation was visible at the seafloor. The instrument was recovered on 30 September 2010 by connecting it to the ship’s wire using the ROV *Genesis* (U Ghent).

### Short sediment temperature lance

*In situ* temperature data from shallow sediment depths at the active centre of the HMMV were obtained using a short temperature lance. The instrument consisted of eight temperature sensors placed 10 cm apart at one end of a 60-m-long thermistor chain. The other end of the cable was connected to a data logger mounted on the observatory frame. The data logger contained an additional pressure sensor (see above). Attached to a metal rod with a few centimetres distance to the rod, the end of the thermistor chain was inserted vertically into the sediment by an ROV. Both the thermistor chain and the data logger were manufactured by RBR Ltd., Canada and calibrated to a precision of ±1 mK prior to the deployment cruise. Temperature readings were recorded at a sampling interval of 20 min.

### Seabed thermistor chain

Seabed temperature across the central area was monitored using a 100-m-long thermistor chain. Equipped with 24 sensor nodes spaced 4 m apart, the thermistor chain covered a 92-m-long transect line from the observatory frame across the centre. It was deployed as a spool attached to the observatory frame and laid out on the seabed by ROV *QUEST* (MARUM). The data logger remained on the observatory frame. Both the thermistor chain and the data logger were manufactured by RBR Ltd. and calibrated to a precision of ±1 mK prior to the deployment cruise. Temperature readings were recorded at a sampling interval of 20 min. All channels functioned without failure throughout the observation.

### Chemistry loggers

The six chemistry loggers, manufactured by RBR Ltd., were each equipped with a pH, an O_2_ and an oxygen reduction potential (ORP) sensor. The sensors were calibrated 1 day before deployment. The loggers were connected by a cable to a central logger on the frame to which the data were mirrored. A manufacturing mistake caused flooding of five of these loggers. From the surviving instrument positioned 50 m south of the frame in the active centre, only the pH data could be extracted. The signals were disturbed by drift and occasional burying into the sediments. Raw pH data, as deposited in the Pangaea database, were for periods of interest corrected by second-order polynomials to approximate bottom-water values of pH 7.8 as reference^8^,^28^.

### AUV surveys

The AUV *Sentry* was operated by the National Deep Submergence Facility team from Woods Hole Oceanographic Institution, USA (http://www.whoi.edu/main/sentry). Its navigation system uses a doppler velocity log and inertial navigation system, aided by acoustic navigation systems (USBL or Long Baseline system (LBL)). A Reson 7125 400 khz multibeam sonar was used to obtain a micro-bathymetric map, by surveying in bottom-following mode at a fixed target altitude of 20 m along survey lines spaced at 50 m offset intervals. Photographs of the seafloor were collected routinely at altitudes of 3.5–5 m off bottom using a 1,000 × 1,000-pixel down-looking digital still camera.

### *In situ* mapping of water-column methane and carbon dioxide

Besides gas flare mapping with the ship’s echosounder (Supplementary Fig. 7), the TETHYS underwater mass spectrometer was operated as payload aboard the *Sentry* AUV. This membrane inlet mass spectrometer has a response time of ~10 s^27^ and minimum limits of detection to <1 part-per-billion on a mole fraction basis^28^. For survey operations of the HMMV, the mass spectrometer was configured to record ion peak measurements in a selected ion-monitoring mode, continuously cycling through ion peak measurements with an acquisition cycle time of ~30 s. Sample water was introduced to the mass spectrometer by means of a 10-cm length of 6-mm diameter stainless steel tubing with an upturned sample inlet to avoid trapping gas bubbles that conducted sample water from ~2 cm away from the AUV’s portside exterior into the mass spectrometer’s sample port inlet. An integrated conductivity temperature and depth sensor (SeaBird FastCat49 CTD) connected in series to the mass spectrometer’s sample port exhaust provided continuous sample flow into the inlet at a rate of ~2 cm s^−1^. Sample water temperature, salinity, water-column pressure and UTC time were recorded by the mass spectrometer at the start and end of each acquisition cycle. Ion peak and physical water parameters were then merged with concurrent AUV navigation position estimates (latitude, longitude and altitude) to generate geo-referenced dissolved methane (*m*/*z* 15) and aqueous carbon dioxide (*m*/*z* 44) measurements (Supplementary Fig. 6). Methane and carbon dioxide concentrations were recorded while the AUV maintained an altitude of 5 m or less above the seafloor during three grid surveys that were used to generate spatial maps of benthic water-column methane and carbon dioxide anomalies.

## Author contributions

D.d.B. with the LOOME team conceived and designed the experiments. All co-authors performed the experiments. T.F., A.B., D.R.Y. and D.d.B. analysed the data. J.B., K.O., F.W. and R.C. contributed observations. T.F., A.B., C.G. and D.d.B. co-wrote the paper with input from all co-authors.

## Additional information

**How to cite this article**: Feseker, T. *et al*. Eruption of a deep-sea mud volcano triggers rapid sediment movement. *Nat. Commun.* 5:5385 doi: 10.1038/ncomms6385 (2014).

## Supplementary Material

Supplementary Information Supplementary Figures 1-8, Supplementary Tables 1-4, Supplementary Notes 1-2 and Supplementary Reference

## References

[b1] Wallmann K., Drews M., Aloisi G. & Bohrmann G. Methane discharge into the Black Sea and the global ocean via fluid flow through submarine mud volcanoes. Earth Planet. Sci. Lett. 248, 544–559 (2006).

[b2] Kaul N., Foucher J.-P. & Heesemann M. Estimating mud expulsion rates from temperature measurements on Håkon Mosby Mud Volcano, SW Barents Sea. Mar. Geol. 229, 1–14 (2006).

[b3] Berndt C. Focused fluid flow in passive continental margins. Phil. Trans. R. Soc. A 363, 2855–2871 (2005). 16286294 10.1098/rsta.2005.1666

[b4] Olu-Le-Roy K. . Cold seep communities in the deep eastern Mediterranean Sea: composition, symbiosis and spatial distribution on mud volcanoes. Deep Sea Res. Part 1 51, 1915–1936 (2004).

[b5] Felden J., Wenzhoefer F., Feseker T. & Boetius A. Transport and consumption of oxygen and methane in different habitats of the Hakon Mosby Mud Volcano (HMMV). Limnol. Oceanogr. 55, 2366–2380 (2010).

[b6] Boetius A. . A marine microbial consortium apparently mediating anaerobic oxidation of methane. Nature 407, 623–626 (2000). 11034209 10.1038/35036572

[b7] Niemann H. . Novel methanotrophic communities of the Haakon Mosby mud volcano and their role as methane sink. Nature 443, 854–858 (2006). 17051217 10.1038/nature05227

[b8] de Beer D. . In situ fluxes and zonation of microbial activity in surface sediments of the Håkon Mosby Mud Volcano. Limnol. Oceanogr. 51, 1315–1331 (2006).

[b9] Milkov A. V., Sassen R., Apanasovich T. V. & Dadashev F. G. Global gas flux from mud volcanoes: a significant source of fossil methane in the atmosphere and the ocean. Geophys. Res. Lett. 30, 1037–1043 (2003).

[b10] Leifer I., Luyendyk B. P., Boles J. & Clark J. F. Natural marine seepage blowout: contribution to atmospheric methane. Glob. Biogeochem. Cycles 20, GB3008 (2006).

[b11] Luff R. & Wallmann K. Fluid flow, methane fluxes, carbonate precipitation and biogeochemical turnover in gas hydrate-bearing sediments at Hydrate Ridge, Cascadia Margin: Numerical modeling and mass balances. Geochim. Cosmochim Acta 67, 3403–3421 (2003).

[b12] Boetius A. & Wenzhöfer F. Seafloor oxygen consumption fuelled by methane from cold seeps. Nat. Geosci. 6, 725–734 (2013).

[b13] Etiope G. & Milkov A. V. A new estimate of global methane flux from onshore and shallow submarine mud volcanoes to the atmosphere. Environ. Geol. 46, 997–1002 (2004).

[b14] Aliyev A. A., Guliyev I. S. & Boelov I. S. Catalogue of Recorded Eruptions of Mud Volcanoes in Azerbaijan (for Period of Years 810–2001) Nafta Press (2002).

[b15] Deville E. & Geurlais S. H. Cyclic activity of mud volcanoes: evidences from Trinidad (SE Caribbean). Mar. Petrol. Geol. 26, 1681–1691 (2009).

[b16] Kopf A. J. Significance of mud volcanism. Rev. Geophys. 40, 1005–1057 (2002).

[b17] Perez-Garcia C., Feseker T., Mienert J. & Berndt C. The Håkon Mosby mud volcano: 330 000 years of focused fluid flow activity at the SW Barents Sea slope. Mar. Geol 262, 105–115 (2009).

[b18] Feseker T., Foucher J.-P. & Harmegnies F. Fluid flow or mud eruptions? Sediment temperature distributions on the Hakon Mosby mud volcano, SW Barents Sea slope. Mar. Geol 247, 194–207 (2008).

[b19] Jerosch K. . Spatial distribution of mud flows, chemoautotrophic communities, and biogeochemical habitats at Haakon Mosby Mud Volcano. Mar. Geol. 243, 1–17 (2007).

[b20] Foucher J.-P. . Changes in seabed morphology, mud temperature and free gas venting at the Hakon Mosby mud volcano, offshore northern Norway, over the time period 2003-2006. Geo-Mar. Lett. 30, 157–167 (2010).

[b21] Sarrazin J. . in *OCEANS '07 IEEE, Aberdeen, UK*. TEMPO: a new ecological module for studying deep-sea community dynamics at hydrothermal vents. Proceedings No. 061215-061042.

[b22] Nouze H. Vicking cruise report: Cold Seeps on the Norwegian Margin http://www.fiskeridir.no/english/fisheries/marine-scientific-research/soekere-2006/0106/cruise-information-pourquoi-pas-060123 Ifremer, Brest (2006).

[b23] Pape T., Feseker T., Kasten S., Fischer D. & Bohrmann G. Distribution and abundance of gas hydrates in near-surface deposits of the Hakon Mosby Mud Volcano, SW Barents Sea. Geochem. Geophys. Geosyst. 12, Q09009 (2011).

[b24] Damm E. & Budéus G. Fate of vent-derived methane in seawater above the Håkon Mosby Mud Volcano (Norwegian Sea). Mar. Chem. 82, 1–11 (2003).

[b25] Sauter E. J. . Methane discharge from a deep-sea submarine mud volcano into the upper water column by gas hydrate-coated methane bubbles. Earth Planet. Sci. Lett. 243, 354–365 (2006).

[b26] Nauhaus K., Albrecht M., Elvert M., Boetius A. & Widdel F. In vitro cell growth of marine archaeal-bacterial consortia during anaerobic oxidation of methane with sulfate. Env. Microbiol. 9, 187–196 (2007). 17227423 10.1111/j.1462-2920.2006.01127.x

[b27] Feseker T. . Eruption of the Håkon Mosby mud volcano recorded by the long-term observatory on mud-volcano eruptions (LOOME) between 2009 and 2010. Pangaea doi:10.1594/PANGAEA.830324 (2014).

[b28] Lichtschlag A., Felden J., Brüchert V. & Boetius A. & de Beer, D. Geochemical processes and chemosynthetic primary production in different thiotrophic mats of the Håkon Mosby Mud Volcano (Barents Sea). Limnol. Oceanogr. 55, 931–949 (2010).

