# Supplementary material for: Eruption of a deep-sea mud volcano triggers rapid sediment movement
Source: Nat Commun. 2014 Nov 11;5:5385. doi: 10.1038/ncomms6385 (PMC4242465; doi:10.1038/ncomms6385)
Supplement: Supplementary Information — Supplementary Figures 1-8, Supplementary Tables 1-4, Supplementary Notes 1-2 and Supplementary Reference [file ncomms6385-s1.pdf]

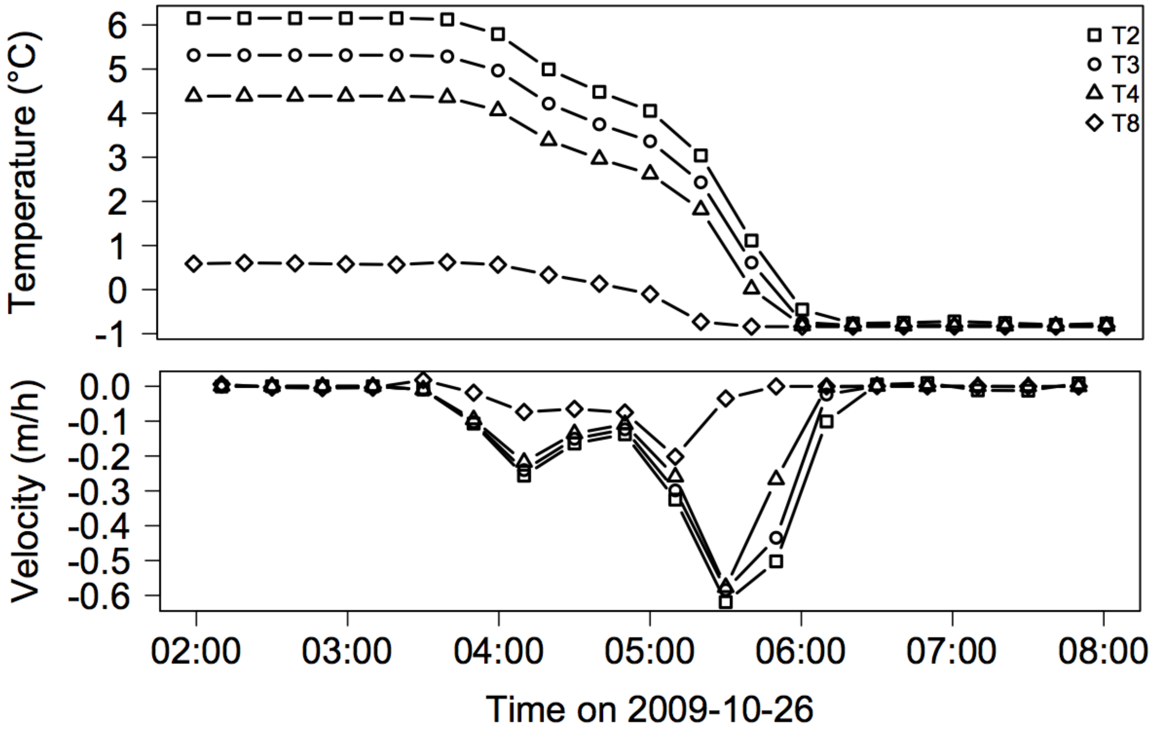

Supplementary Figure 1: Temperatures recorded by the short temperature lance. The short temperature lance was placed at the warm center of the mud volcano and connected to the LOOME observatory frame by a cable. The simultaneous decrease of temperature to bottom water values for all sensors within 3 hours (ca. 03:30 – 06:30) on 2009-10-26 indicates that the lance was pulled out as the surrounding sediment moved away from the observatory frame. Assuming a stable sediment temperature profile, the vertical velocity of the lance during the pull-out can be reconstructed from the temperature changes at the sensors T2, T3, T4, and T8, which started at depths of ca. 0.75, 0.65, 0.55, and 0.15 m, respectively, and were pulled upwards and out of the sediment onto the seafloor.

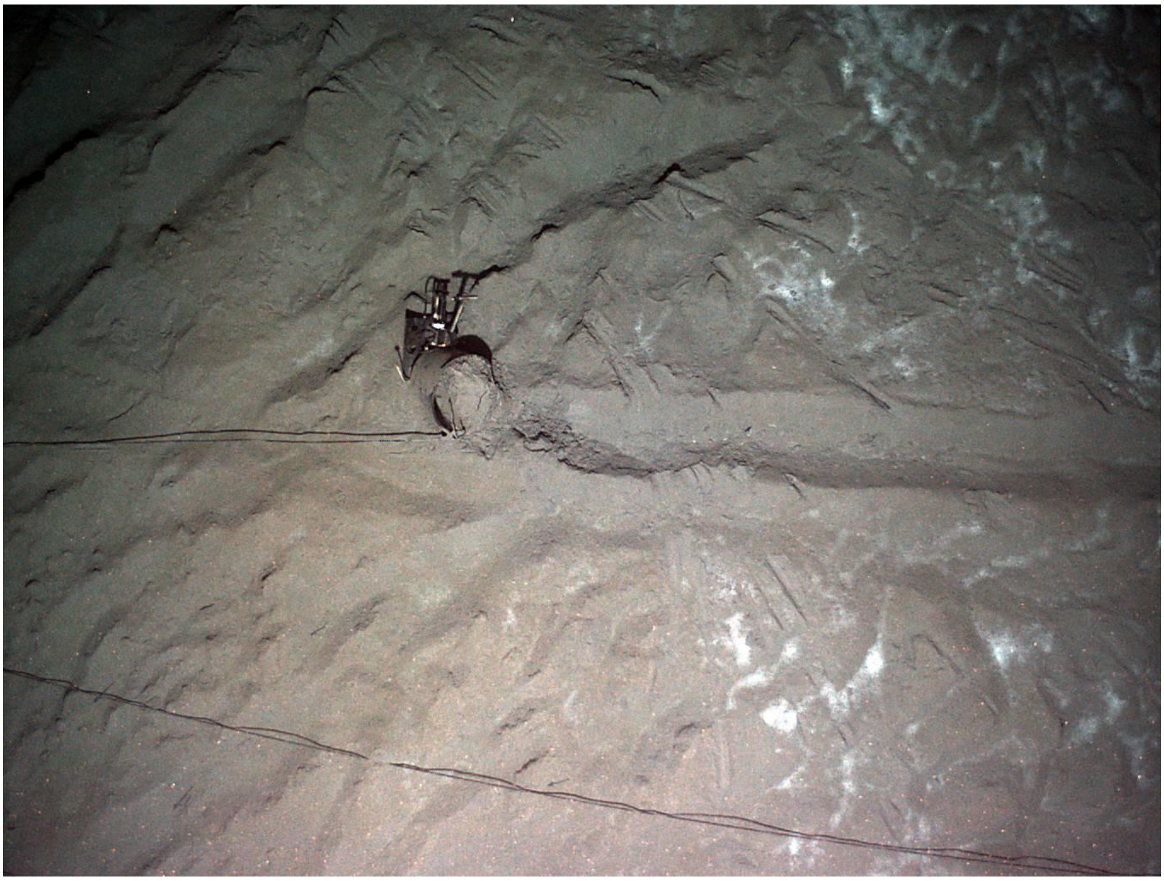

Supplementary Figure 2: Downward-looking Sentry photograph of the pH sensor. The trail runs from the sensor towards the frame. The trail is approximately 35 cm wide. Source: WHOI ABE/Sentry team.

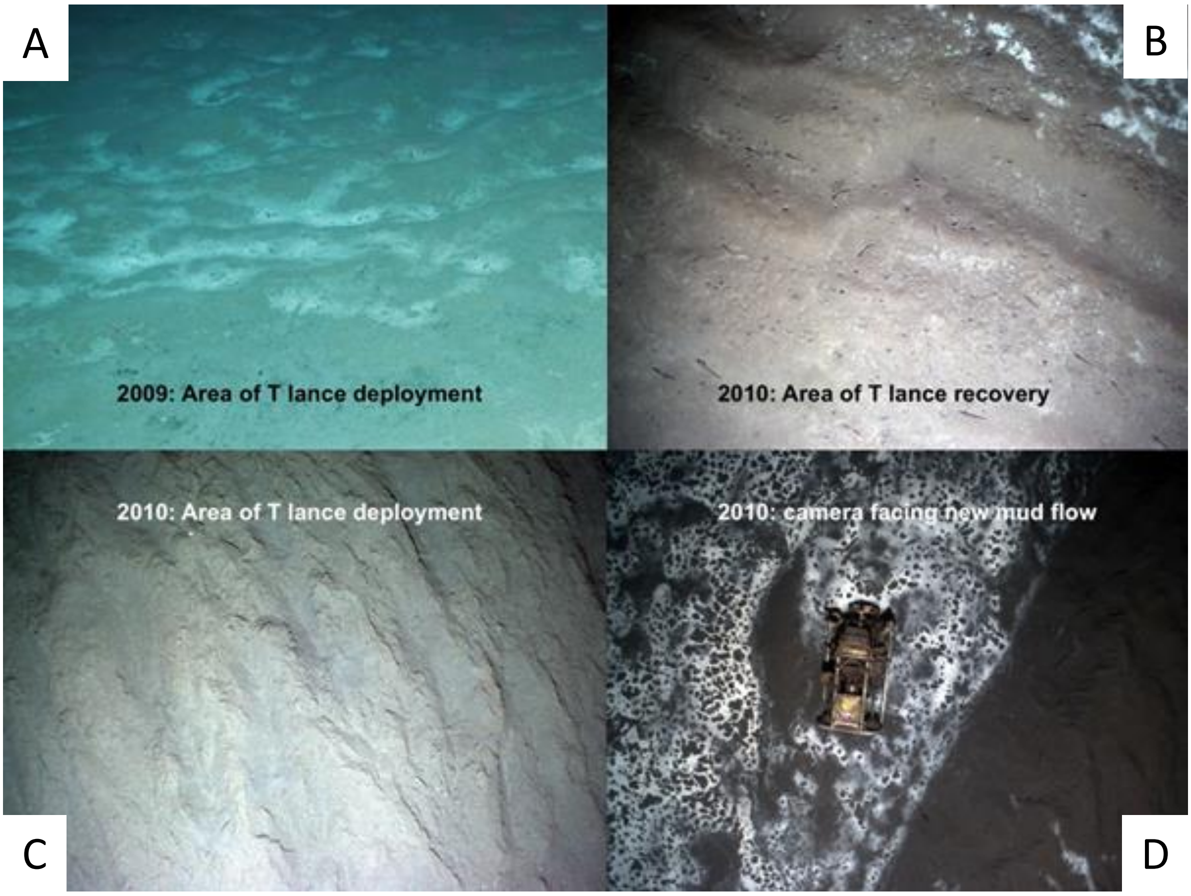

Supplementary Figure 3: Images of the changes in sediment morphologies. Photos are made by forward looking ROV (Fig. 3A) during the LOOME deployment in 2009 and by downward looking SUV during the recovery in 2010 (Fig. 3B-D). Note the gas bubble holes in 2010 around the T lance position (B), and the strongly altered seafloor morphology of the former T lance position in 2010 with troughs and cracks (C), as at the new mud flow recorded by the camera (D). The width of Figs. 3A-C is roughly 2 m, of Fig. 3D 6-7 m. Sources: Fig. A, copyright MARUM University of Bremen; Figs B-D, WHOI ABE/Sentry team.

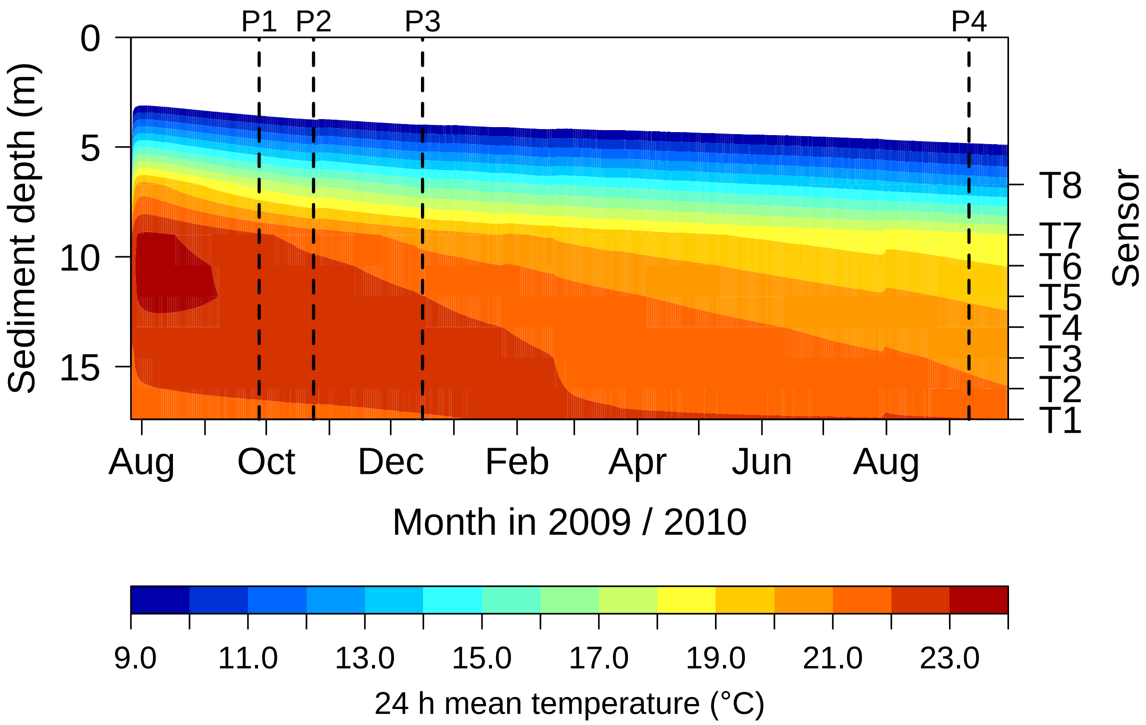

Supplementary Figure 4: Temperature dynamics in the upper 18 m of sediment. The temperatures were measured along the shaft of the 15 m long temperature lance with 8 sensors distributed along its shaft. This is the lance that moved 165 m during its deployment. P1 through P4 refer to the 4 major uplifting events.

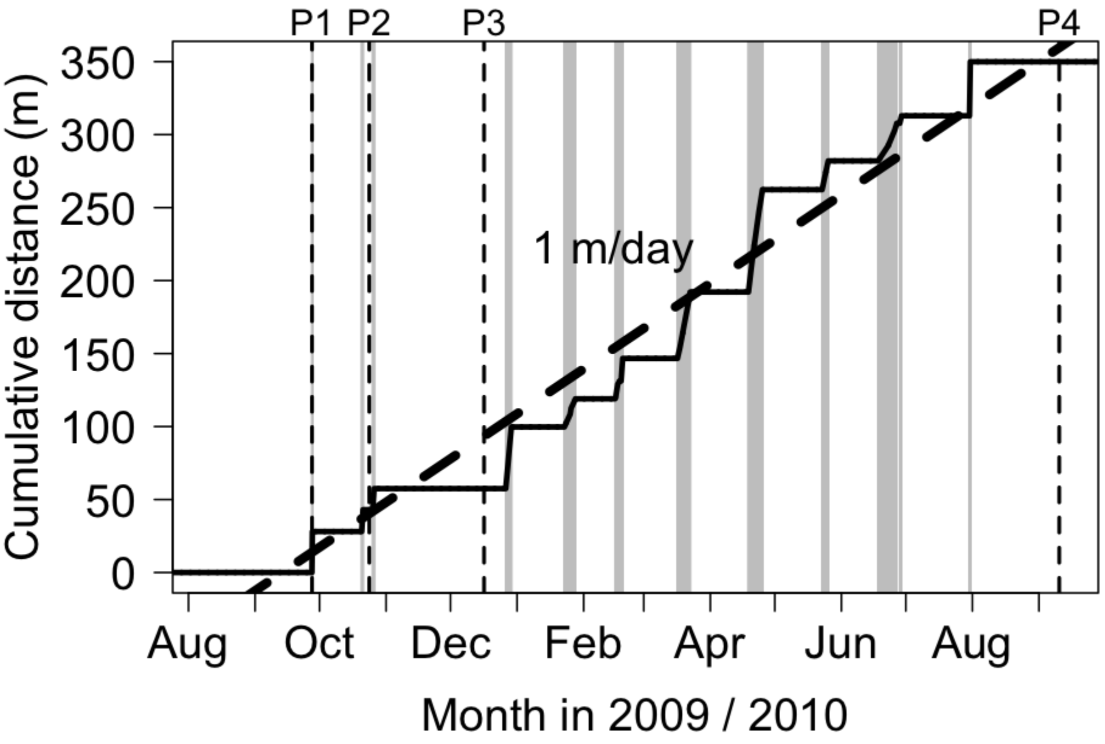

Supplementary Figure 5: Cumulative lateral sediment movement along the thermistor chain.

The solid black line shows the cumulative distance moved by the sediment, according to traces of warm sediment identified in the seabed temperature time series. Periods of movement are shaded in grey. The thick dashed line indicates a trend of 1 m per day for the entire duration of the observation. The vertical dashed lines mark the times of sudden uplift events. P1 through P4 refer to the 4 major uplifting events.

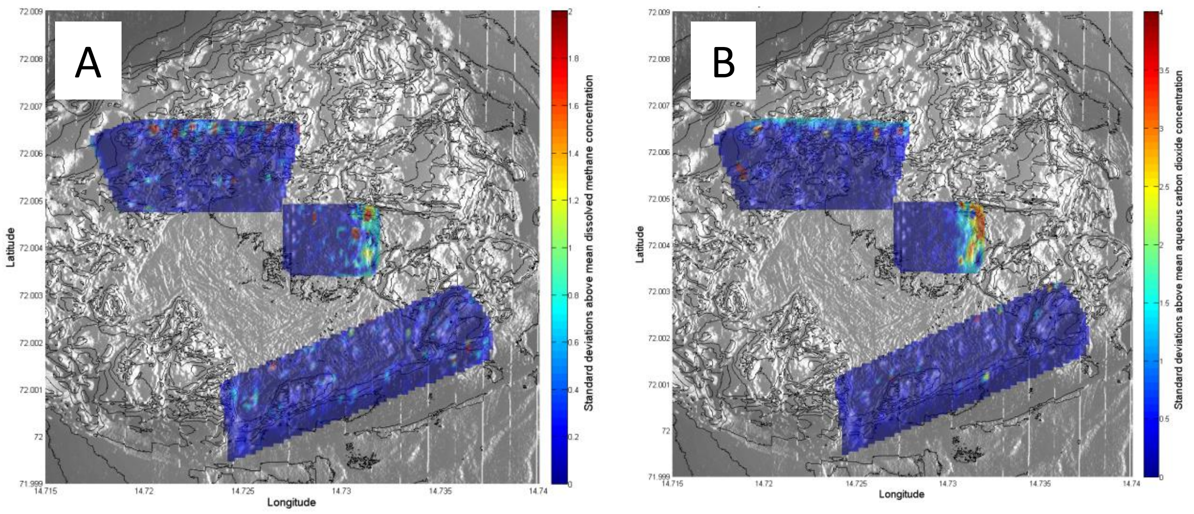

Supplementary Figure 6: Distribution of methane (A) and CO_2 (B) at 5 m above the seafloor.

The gases were detected by the UW-MS mounted on the AUV Sentry. Both flares are most prominent in the most active area. The slight difference between the positions of both flares is explained by the different interactions with water of the two compounds. CO_2 dissolves faster, thus methane travels further in bubbles.

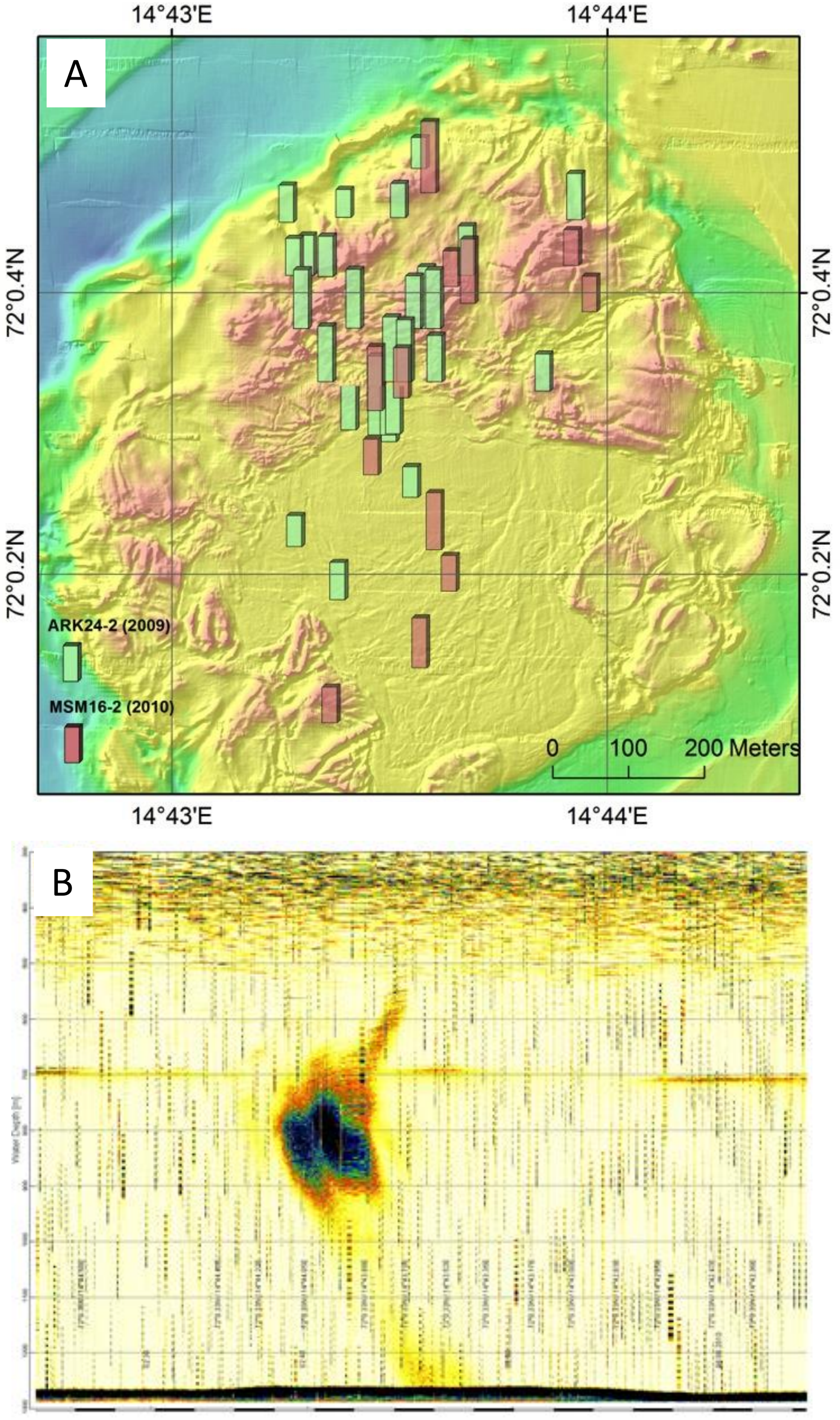

Supplementary Figure 7: **Gas flares recorded by hydroacoustics in 2009 and 2010.** A) Distribution of gas flares recorded by the shipboard subbottom profiler (Atlas Parasound P-70, 20 kHz frequency). In 1250 m water depth the echo sounder's acoustic footprint is approximately 90 m. B) Echogram plot of a single gas

flare (echogram y-axis: 300-1300 m depth, lines in 100 m interval, x-axis: ~1000 m, each bar 50 m)

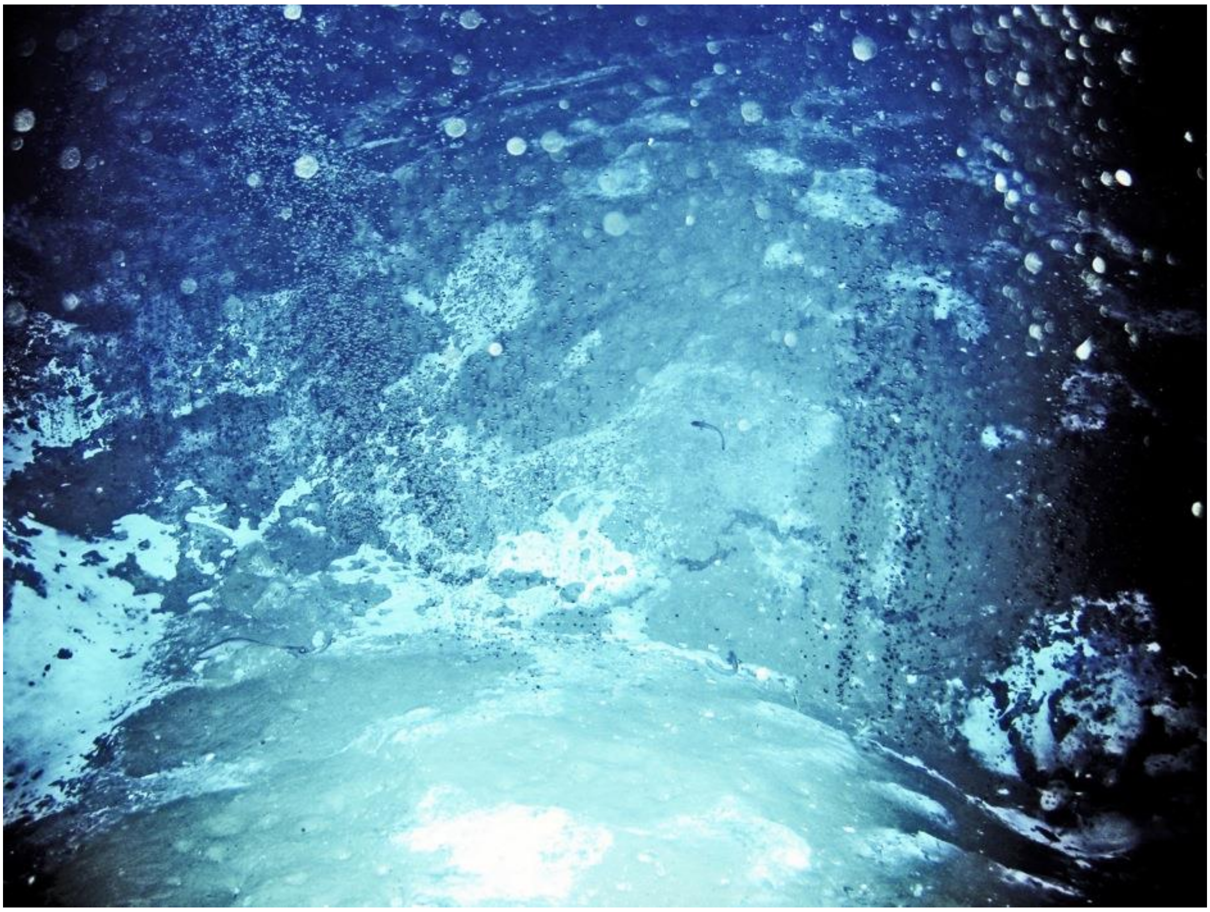

Supplementary Figure 8. Observation of vigorous gas venting from the seafloor close to the LOOME observatory (source: ROV Genesis, University Gent).

Supplementary Table 1: Time and Positions of the LOOME observatory components deployed in 2009 and recovered in 2010

**Components**	**N**	**E**	**deployment/ recovery date (UTC)**	**deployment mode**	**recovery mode**
**LOOME frame (MPI)**	72° 00.324'	014° 43.561'	24.7.2009/ 28.9.2010	ROV/winch	ROV/winch
**T-String 100m (GEOMAR)**	72° 00.272'	014° 43.608'	25.7.2009/ 28.9.2010	ROV	ROV/winch
**T-Stick Loome (GEOMAR)**	72° 00.299'	014° 43.590'	25.7.2009/ 28.9.2010	ROV	ROV/winch
**pH Microsensor string (MPI)**	72° 00.294'	014° 43.590'	25.7.2009/ 28.9.2010	ROV	ROV/winch
**Camera AIM (Ifremer)**	72° 00.312'	014° 43.626'	26.7.2009/ 28.9.2010	ROV	ROV/winch
**T-Lance Loome (Ifremer)**	72° 00.246'<br>72° 00.156'	014° 43.608'<br>014° 43.611'	27.7.2009<br>30.9.2010	Winch	 ROV/winch

Supplementary Table 2: Rapid uplift events recorded by a pressure sensor at the LOOME observatory frame.

	Date	Time UTC	Uplift	Duration	Max. rate of uplift
**P1**	2009-09-27	12:40	0.6 m	3.5 h	0.4 m/h
**P2**	2009-10-24	04:20	0.3 m	1 h	0.7 m/h
**P3**	2009-12-16	15:00	0.3 m	3 h	0.2 m/h
**P4**	2010-09-10	11:00	0.15 m	40 min	0.4 m/h

Supplementary Table 3: Turbidity events recorded by the RCM at the LOOME frame.

**Period**	**Current speed Mean (cm sec^{-1})**	**Current direction Mean (degree)**	**Turbidity events > NTU 1**
T1 (25-07-09 – 21-08-09)	5.9	164	14
T2 (22-08-09 – 18-09-09)	7.4	192	15
T3 (19-09-09 – 16-10-09)	5.9	191	5
T4 (17-10-09 – 13-11-09)	6.5	166	1
T5 (14-11-09 – 11-12-09)	5.6	240	2
T6 (12-12-09 – 08-01-10)	7.0	188	0
T7 (09-01-10 – 05-02-10)	6.2	215	14
T8 (06-02-10 – 06-03-10)	5.6	123	1
T9 (07-03-10 – 03-04-10)	5.3	155	2
T10 (04-04-10 – 02-05-10)	5.9	236	47
T11 (03-05-10 – 30-05-10)	5.9	110	1
T12 (31-05-10 – 27-06-10)	5.4	176	0
T13 (28-06-10 – 25-07-10)	5.3	215	7
T14 (26-07-10 – 22-08-10)	3.2	232	11
T15 (23-08-10 – 19-09-10)	5.0	245	1

Background NTU was 0.75.Maximum turbidity peaks observed reached 2 NTU.

Supplementary Table 4: Warm temperature events at the seafloor interpreted as indicators of lateral sediment movement

	Starting time UTC	Duration (h)	Distance from observatory frame (m)	Length (m)	Velocity (m/h)
**1**	27-09-2009 12:12	4.1	28.5	23.29	5.6
**2**	20-10-2009 16:13	14.5	47.9	14.52	1
**3**	25-10-2009 19:07	14.1	28	12.38	0.9
	25-10-2009 20:52	14	12.3	11.48	0.8
	26-10-2009 05:59	3.6	35	5.18	1.4
**4**	26-12-2009 17:22	63.8	13.5	42.76	0.7
**5**	23-01-2010 00:54	124.6	35.4	16.32	0.1
	23-01-2010 05:14	66.6	51.3	9.34	0.1
	25-01-2010 16:53	10.8	24.1	7.99	0.7
**6**	15-02-2010 14:32	35.1	60.1	3.6	0.1
	15-02-2010 21:18	36.1	55.7	3.94	0.1
	15-02-2010 22:33	23.1	11.8	27.68	1.2
	16-02-2010 04:15	37.6	51.5	4.16	0.1
**7**	18-02-2010 11:18	17.3	69.5	14.85	0.9
**8**	16-03-2010 17:10	125.2	13.5	34.1	0.3
	17-03-2010 19:23	115.5	39.8	43.43	0.4
**9**	18-04-2010 18:13	57.5	52.3	29.48	0.5
	20-04-2010 08:17	80.8	41.8	39.83	0.5
	20-04-2010 13:46	112.9	24.2	43.1	0.4
**10**	23-05-2010 01:06	68.9	56.2	19.69	0.3
**11**	18-06-2010 03:23	184.7	43.2	17.1	0.1
	22-06-2010 19:44	96.5	28	19.35	0.2
**12**	28-06-2010 05:14	19.7	38.5	5.06	0.3
**13**	30-07-2010 15:21	10.5	23.1	32.52	3.1
	30-07-2010 16:55	8.6	43.9	38.48	4.5

Supplementary Note 1

Observations of impact of lateral mud movement on sensor placements

The short temperature lance was installed on 2009-07-24 at the HMMV centre, where, based on the mean geothermal gradient[1], we expected the highest level of activity. The lance was inserted vertically by ROV with full penetration. However, when we visually inspected the location on 2010-09-27 immediately prior to recovery, the lance was lying flat on the sediment surface and the cable connecting the lance to its logger on the observatory frame appeared to be fully stretched. On 2009-10-26, two days after the sudden uplift event P2, the temperature signal of all sensors began to decrease at 03:40 UTC and reached bottom water levels at 06:20 UTC (see Supplementary Fig. 1). This can only be explained by the lance being pulled out due to lateral sediment displacement, a process that also caused trail marks (“wakes”) around other sensors too (Supplementary Fig. 2). Assuming a stable sediment temperature profile at this time, the vertical motion of the lance reconstructed by calculating the depths of the sensors from the temperature profile prior to its extraction from sediment yielded a peak upward velocity of around 0.6 m/h (Supplementary Fig. 1). As the extraction of the short T-lance from the seabed was probably a combination of vertical, horizontal, and rotational movements, the reconstructed vertical velocity is a conservative estimate. During the time period between deployment and its extraction the temperature profiles recorded by the short lance documented the continuous cooling of the surface sediments from an initial temperature gradient of nearly 18 °C/m to less than 10 °C/m.

Supplementary Note 2

Observations on turbidity

Interestingly, the gas venting (Supplementary Fig. 6, 7 and 8) did not cause substantial anomalies in the turbidity sensor mounted on the LOOME observatory frame, situated upstream of the vents. Instead, the turbidity signals that can be detected from our time series record were all rather low (<2 NTU) despite being frequent, and appear to only be partially associated with the seafloor fluid flow events that our work has detected (Supplementary Tables 3 and 4).

Supplementary References

1 Foucher, J.-P. *et al.* Changes in seabed morphology, mud temperature and free gas venting at the Hakon Mosby mud volcano, offshore northern Norway, over the time period 2003-2006. *Geo-Mar Lett* **30**, 157-167 (2010).
